# Systematic and mandatory monitoring by the public health authorities – a novel approach to improve infection control and increase patient safety

**DOI:** 10.1186/2047-2994-4-S1-P104

**Published:** 2015-06-16

**Authors:** C Höller, V Lehner-Reindl, B Liebl

**Affiliations:** 1 Hygiene, Bavarian Health and Food Safety Authority, Oberschleissheim, Germany; 2 Bavarian Health and Food Safety Authority, Oberschleissheim, Germany

## Introduction

Although infection control is the main responsibility of hospitals themselves the public health system has its own important role in enhancing patient safety. The increasing threat of multiresistant bacteria and outbreaks of nosocomial infections gave a reason to restructure the tasks and responsibilities of the Bavarian public health service in 2010.

## Objectives

Monitoring of medical institutions had been performed by the public health only when there was a cause of concern. This was considered to be insufficient and to lead to inadequate knowledge in hospital hygiene. Accordingly a multi-step improvement was carried out: a) knowledge in hospital hygiene was enhanced by introducing mandatory yearly training courses and allocating more specific teaching time in the basic training of public health staff, b) a special unit (SEI) for hospital hygiene with the authority to enforce legal actions was founded and c) yearly mandatory, standardized monitoring programs with special focus points are performed by the public health offices and the SEI.

## Methods

The monitoring programs consisted up to 2015 in following topics: quality and amount of infection control staff; operation, emergency and obstetric department; intensive care units (ICU); bone marrow transplantation units; out-patient operative centers; surveillance of nosocomial infections on ICU; management of multi-resistant bacteria and antibiotics; performance of hand hygiene and decontamination of the environment. Public health offices and SEI used during the inspections newly developed check lists to ensure a standardized monitoring protocol. The results were communicated locally, to the Ministry of Health, to medical associations and to professionals in the field.

## Results

The awareness for the problems in hospital hygiene was enhanced tremendously by this multistep program. For instance additional staff for infection control is assigned increasingly. Several training centers started not only new courses but participated also in defining standard teaching courses. The monitoring programs revealed constructional deficiencies, which led to a specifically funded building program.

## Conclusion

All these improvements are important aspects of enhancing patient safety.

## Disclosure of interest

None declared.

